# PNPLA1-Mediated Acylceramide Biosynthesis and Autosomal Recessive Congenital Ichthyosis

**DOI:** 10.3390/metabo12080685

**Published:** 2022-07-26

**Authors:** Fansi Zeng, Wenzhen Qin, Feifei Huang, Pingan Chang

**Affiliations:** 1 Chongqing Key Laboratory of Big Data for Bio-Intelligence, School of Bio-Information, Chongqing University of Posts and Telecommunications, Chongqing 400065, China; zengfansi123@icloud.com (F.Z.); huangff@cqupt.edu.cn (F.H.); 2 Laboratory of Molecular Biology, Chongqing University of Posts and Telecommunications, Chongqing 400065, China; qinwenzhen@cqupt.edu.cn

**Keywords:** PNPLA1, acylceramide, skin barrier, ARCI, gene mutation

## Abstract

The stratum corneum of the epidermis acts as a life-sustaining permeability barrier. Unique heterogeneous ceramides, especially ω-O-acylceramides, are key components for the formation of stable lamellar membrane structures in the stratum corneum and are essential for a vital epidermal permeability barrier. Several enzymes involved in acylceramide synthesis have been demonstrated to be associated with ichthyosis. The function of patatin-like phospholipase domain-containing protein 1 (PNPLA1) was a mystery until the finding that *PNPLA1* gene mutations were involved in autosomal-recessive congenital ichthyosis (ARCI) patients, both humans and dogs. PNPLA1 plays an essential role in the biosynthesis of acylceramide as a CoA-independent transacylase. PNPLA1 gene mutations cause decreased acylceramide levels and impaired skin barrier function. More and more mutations in PNPLA1 genes have been identified in recent years. Herein, we describe the structural and functional specificity of PNPLA1, highlight its critical roles in acylceramide synthesis and skin barrier maintenance, and summarize the PNPLA1 mutations currently identified in ARCI patients.

## 1. Introduction

The skin is composed of the outer epidermis, the dermis, and subcutaneous tissue, from the outside to the inside. There are four layers in the stratified epidermis from the inside to the outside, including the stratum basale (SB), the stratum spinosum (SS), the stratum granulosum (SG), and the stratum corneum (SC). The SC functions as a life-sustaining permeability barrier that suppresses excess evaporation of water and the loss of small molecules from the skin, while also serving as an antimicrobial barrier quelling the penetration of exogenous molecules, allergens, and microorganisms into the skin. Unique heterogeneous ceramides, especially ω-O-acylceramides, are key components for the formation of stable lamellar membrane structures in the SC and are essential for a vital epidermal permeability barrier. The patatin-like phospholipase domain-containing protein 1 (PNPLA1) has been identified as an essential transacylase for acylceramide biosynthesis to maintain the epidermal permeability barrier [1]. In recent years, more and more mutations of the PNPLA1 gene have been found to be linked to autosomal-recessive congenital ichthyosis (ARCI). Here, we summarize the structural and functional specificity of PNPLA1 and emphasize its essential role in acylceramide biosynthesis and maintenance of the skin barrier, also pointing out the PNPLA1 mutations found in ARCI patients.

## 2. The Structure, Expression and Subcellular Location of PNPLA1

**Figure 1:**
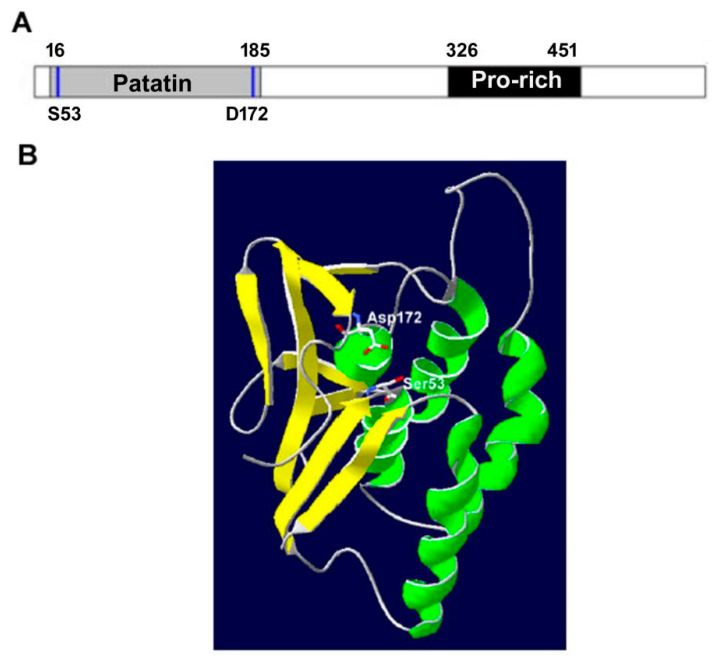
Protein domains of the human PNPLA1. (**A**) Patatin domain (aa 16-185) and a proline-rich hydrophobic region (aa 326–451) were present in the N- and C-terminal regions, respectively. S53 and D172 represent the conserved catalytic residues of serine and aspartate in the patatin domain. (**B**) Molecular model of the patatin domain of human PNPLA1. Regions predicted to fold as β-sheets and α-helices are shaded yellow and green, respectively. Side chains of the active sites, Asp172 and Ser53, are rendered in stick format and the atoms colored using standard Corey, Pauling and Kultun (CPK).

The full length of the human *PNPLA1* gene is 38.14 kb and it contains eight exons at chromosome 6p21.31 [2,3]. The human PNPLA1 protein consists of 532 amino acids and contains a patatin domain (residues 16-185) and a proline-rich hydrophobic region (residues 326–451), which is unique to PNPLA1 and whose function remains unknown (Figure 1A) [3]. In the patatin domain, the conserved serine lipase pattern (GXSXG) (GTSAG, residues 51–55) and the consensus DGG-motif contain the active sites Ser53 and Asp172, respectively, which constitute the catalytic dyad of PNPLA1 [3]. The patatin domain in human PNPLA1 shares a conserved core module with mammalian lipase containing three β-chains and one α-helix chain [3]. Molecular modeling shows that the patatin domain is characterized by a three-layer α/β/α architecture employing a catalytic Ser–Asp catalytic dyad. The catalytic Ser53 is located at the tight transition between β-folding and α-helix within a nucleophilic elbow, whereas the catalytic Asp172 is within the upper spatial position (Figure 1B) [3]. Moreover, a highly conserved extended patatin domain (residues 1–288) is proposed to probably represent an essential functional unit for PNPLA1 to exhibit its enzymatic activity [4].

In adult dog and mouse tissues, PNPLA1 messenger RNA (mRNA) is the most abundant in the skin [2,5,6,7]. The *Pnpla1* gene in mice also has a certain amount of expression in other surface lining tissues, such as the dermis, tongue and stomach [5,6]. In healthy human skin, a stronger expression of PNPLA1 is observed in the granular layer of the epidermis and in the eccrine sweat gland cells of the dermis [2]. A more pronounced expression of PNPLA1 in the upper epidermal layers and the lower layers of the cornified layer is also detected in the region of keratin filament bundles [2]. PNPLA1 is detected by immunohistochemistry in the border region between the nucleated SGs and denucleated SCs in the skin of neonatal mice [7]. The localization of PNPLA1 in the newborn and adult mouse epidermis is essentially the same as that in human skin [7]. Ca^2+^ treatment resulted in a marked induction of PNPLA1 in mouse and human differentiated keratinocytes, suggesting a specific role of PNPLA1 in highly differentiated keratinocytes in the uppermost layer of the SG [7].

The expression of PNPLA1 alone was distributed in the cytoplasm, except in nuclei, endoplasmic reticulum (ER), and lipid droplets (LDs) [5,8,9]. However, PNPLA1 protein colocalized with LDs in skin fibroblasts from healthy individuals and ARCI patients [10]. The interaction of PNPLA1 with LDs may be mediated by its carboxy-terminal region [3]. When the co-expression of PNPLA1 with α/β-hydrolase domain-containing protein 5 (ABHD5) occurred, PNPLA1 was recruited to intracellular LDs, which was observed in cells with low expression of PNPLA1 and ABHD5 [8,9]. This may represent a transitional stage. Different levels of PNPLA1 and ABHD5 caused morphological changes in LDs. PNPLA1 was dispersed throughout the cytoplasm in cells with high PNPLA1 and ABHD5 expression and the disappearance of LDs [9]. These LDs might be incorporated into the ER [9]. The localization of PNPLA1 to LDs in an ABHD5-dependent manner implies an interaction between PNPAL1 and ABHD5. 

## 3. PNPLA1 Acts as a CoA-Independent Transacylase for Acylceramide Biosynthesis 

The permeability barrier in the skin of terrestrial mammals not only suppresses excess evaporation of water and loss of small molecules from skin, but also quells the penetration of exogenous molecules, allergens, and microorganisms into skin as an antimicrobial barrier [10]. The permeability barrier acts in part as a thermal barrier in the SC to prevent hypothermia due to excess water evaporation [10]. In the SC, the lipid lamellae play a central role in skin barrier function and are mainly composed of ceramides, cholesterol, and fatty acids (FAs), among which unique heteroceramides are key components [11]. Of the various ceramides, epidermis-specific acylceramides (ω-O-acylceramides) are specialized lipids for skin barrier formation [12].

As with all ceramides’ biosynthetic pathways, the *de novo* biosynthesis of acylceramide was initiated by serine palmitoyltransferase (SPT), which condensed serine with palmitoyl-CoA to produce 3-ketodihydrosphingosine [12]. The 3-ketodihydrosphingosine was then reduced to dihydrosphingosine, catalyzed by 3-ketodihydrosphingosine reductase (KDSR) [13]. Fatty acid elongase ELOVL1 extended long-chain (LC) acyl-CoA to C26, which was further elongated to C30-C36 ultra-long-chain (ULC) acyl-CoA by ELOVL4 [14,15]. Ultra-long-chain fatty acids (ULCFAs) are unique to acylceramides. After removing CoAs from ULC acyl-CoA, ULCFAs were ω-hydroxylated by the action of CYP4F22, a member of the cytochrome P450 family, to produce ω-OH ULCFAs [16,17]. Next, ω-OH ULCFAs were converted to ω-OH ULC acyl-CoAs by fatty acid transporter protein 4 (FATP4) as an acyl-CoA synthetase [18,19]. The de novo synthesis of dihydrosphingosine as the primary long-chain base (LCB) donor and ω-OH ULC acyl-CoAs as the fatty acyl donor were incorporated by ceramide synthase CERS3 for ω-OH ceramide synthesis [20]. 

In addition to the two hydrophobic chains in normal ceramides, acylceramides have an additional hydrophobic chain, linoleic acid. The ester bond formation between ω-OH ceramide and linoleic acid is the final step of acylceramide production. In 2017, PNPLA1 was demonstrated to be responsible for the final step of acylceramide production as a CoA-independent transacylase [21]. PNPLA1 used triacylglycerol (TG), but not linoleoyl-CoA, as the donor of linoleic acid [21]. The long-chain acyl-CoA synthetase 1 (Acsl1) was recently shown to be essential for the conversion of linoleic acid to linoleoyl-CoA for acylceramide biosynthesis [22]. Linoleoyl-CoA was then catalyzed by diacylglycerol acyltransferase 2 (DGAT2) for TG biosynthesis as a linoleic acid reservoir [23]. The role of PNPLA1 in acylceramide biosynthesis was demonstrated in *Pnpla*1-deficient mice. The amount of acylceramides and their glucosylated derivative acylglucosylceramides were barely detectable in global *Pnpla1*^−/−^ mice [6,7,24]. In contrast, the proposed precursor lipids for acyl(glucosyl)ceramide synthesis, ω-OH-ceramides and the glucosylated derivatives as well as ω-OH ULCFAs substantially accumulated in *Pnpla1*-deficient epidermis [6,7,24]. The same results were found in the epidermis of keratinocyte-specific *Pnpla1*-ablated mice [7]. At the same time, a significant albeit modest increase in free linoleic acid was observed in *Pnpla1*-deficient mice [7]. Therefore, PNPLA1 is required for epidermis acylceramide biosynthesis in a cell-autonomous manner. 

In addition, the epidermal levels of (O-acyl)-ω-hydroxy fatty acid (OAHFA) species, particularly (O-linoleoyl)-o-hydroxy FA (OLHFA), were markedly decreased with a reciprocal increase in corresponding ω-hydroxy ULCFAs in *Pnpla1*^−/−^ mice [7,24]. Interestingly, the most recent findings demonstrate that adipose triglyceride lipase, namely PNPLA2, acts as a transacylase in mammals to catalyze the formation of ester bonds in fatty acid esters of hydroxy fatty acids (FAHFAs) [25]. These results suggest that PNPLA1 may also be involved in the linoleoyl ω-O-esterification of free ω-hydroxy ULCFAs, although OAHFAs may be generated indirectly after the degradation of acylceramides. 

**Figure 2:**
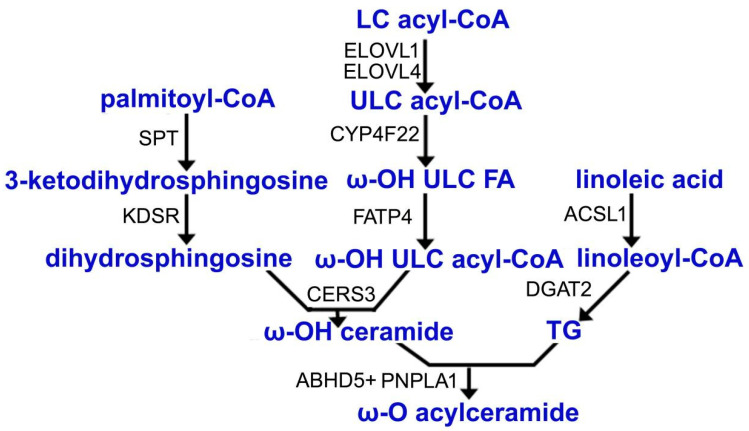
Acylceramide biosynthesis pathway. Metabolites and final products are marked in blue and the catalytic enzymes of each reaction are shown in black. The ω-O-acylation prior to ceramide synthesis remains uncertain. This figure tentatively shows that ω-O-acylation occurs after ω-OH ceramide production.

As mentioned above, ABHD5 recruited PNPLA1 to LDs [8,9]. This effect was mediated by the ABHD5–PNPLA1 interaction [9]. The expression of PNPLA1, but not ABHD5, increased acylceramide levels. The levels of acylceramides were further elevated in PNPLA1 and ABHD5-coexpressing cells, indicating that ABHD5 enhanced PNPLA1-dependent acylceramide production [8,9]. However, the levels of TG as a linoleic acid reservoir were not decreased by PNPLA1 and ABHD5 expression [9]. To compensate for the shortage of acylceramide in *Pnpla1*-deficient epidermis, the expression levels of genes related to acylceramide synthesis, such as *Elovl4*, *Cyp4f39* (a mouse ortholog of human CYP4F22), *Cers3* and *Abhd5*, were significantly upregulated [6,7]. When *Fatp4* was deleted in the skin, the mRNA levels of *Pnpla1* were decreased [19]. However, the deletion of *Acsl1* in mice did not affect the expression of the P*npla1* gene [22]. These changes may be an adaptive mechanism of acylceramide biosynthesis. A summary of the biosynthesis pathways of acylceramides is shown in Figure 2. 

## 4. PNPLA1 Is Essential for Skin Barrier Function

The skin barrier of terrestrial mammals serves a vital function by preventing against internal water and electrolyte loss as well as the external penetration of harmful substances and pathogenic microorganisms [26]. Impaired skin barrier function can cause or exacerbate skin diseases, including dry skin, ichthyosis, psoriasis, and atopic dermatitis [26]. The skin barrier is primarily formed in the SC, the outermost cell layer of the epidermis, in which multilayered lipids (lipid lamellae) fill intercellular spaces [26]. Ceramides, including acylceramides, are produced from the upper layer of the SS to the SG, and are converted to glucosylceramides (acyl-glucosylceramides) or sphingomyelins as the precursor lipids of lipid lamellae, and stored in the lamellar bodies [12]. Lamellar bodies are fused with the plasma membrane at the interface of SG and SC, and the precursor lipids are secreted into the intercellular space in the SC. They are converted to ceramides/acylceramides, together with an appropriate ratio of cholesterol and FAs, to form lipid lamellae [12]. Keratinocytes, the most numerous epidermal cells, proliferate in the stratum basale and migrate outward while differentiating into cell-layer-specific cells. The terminally differentiated keratinocytes, corneocytes, in the SC are surrounded by extracellular lipid lamellae. A cornified envelope (CE) forms on the periphery of the upper layers of epidermis. The CE is formed by the crosslinking of proteins such as involucrin, envoplakin, periplakin, loricrin, and small proline-rich proteins by transglutaminase under the plasma membrane [27]. The lipid bilayer of the keratinocytes’ plasma membrane is replaced with a monolayer of protein-bound Cers, where ω-OH Cers are crosslinked with CE proteins. The protein-bound ceramide-containing membrane structure is named the corneocyte lipid envelope (CLE), and it constructs the connection between corneocytes and lipid lamellae [28].

In 2017, three laboratories independently reached the same conclusion from *Pnpla1*-deficient mice that PNPLA1 is essential for epidermal acylceramide biosynthesis, and that the decrease in or absence of acylceramide biosynthesis impaired the barrier function of the skin and led to ichthyosis [6,7,24]. *Pnpla1*-deficient mice died shortly after birth from excessive epidermal dehydration, showing disruption of the inner–outer epidermal permeability barrier. On the other hand, *Pnpla1*^−/−^ pups showed intense toluidine blue staining, while wild-type littermates excluded the dye, indicating a defect in the outer–inward permeability barrier in the skin of *Pnpla1*-deficient mice. Thus, PNPLA1 is required for epidermal permeability barrier function. The *Pnpla1*-deficient mouse skin is characterized by hyperkeratosis, with a tightly packed SC structure, mild acanthosis with increased SS cell layers, and reduced epidermal rete ridges and keratohyalin granules [6,7]. The typical features of severe ichthyosis, including the extensive loss of the highly organized lipid lamella structure in the SC, abnormal secretion of lamellar granule contents at the SG–SC interface, and unusual lipid aggregates within corneocytes, were observed in Pnpla1-knockout mice [7,23]. A diminished concentration of acylceramides alters or misses the long periodicity lamellar phase (LPP) with repeat distances of approximately 11–13 nm for typical multilamellar lipid assemblies in SC, and increases permeability [29]. The loss of acylceramide linoleate moiety in *Pnpla1*^−/−^ mice disrupted the lipid lamellae pairing in lamellar bodies and the LPP pattern of the SC lipids [30]. In addition, the linoleate moiety in acyl(glucosyl)ceramides is required for attaching to corneocyte proteins to form the CLE [28,31]. In the absence of ω-O-acylceramides, lipids of *Pnpla1*^−/−^ epidermis were prone to separation into LPP and medium lamellar phase (MLP) with a 10.8 nm-repeat distance, leading to the diminished barrier properties [30].The CLE was absent in *Pnpla1*^−/−^ epidermis due to the lack of ω-O-acyl (glucosyl) ceramides, but not the metabolically active enzymes related to the CLE formation [6]. After the topical application of epidermal lipids from WT mice, *Pnpla1*-deficient skin is capable of rebuilding the CLE [6]. A keratinocyte-specific deletion of Pnpla1 mice also exhibited the impaired skin barrier function [7]. 

PNPLA1 is also involved in the proliferation and differentiation of epidermal keratinocytes. The expression of late keratinocyte differentiation and corneocyte envelope assembly markers including filaggrin, involucrin and loricrin was reduced in *Pnpla1*-deficient mice skin, while the levels of keratinocyte-proliferation markers such as keratin 6 and Ki67 were elevated [6,7,24]. The less monomeric filaggrin resulted from the disruption of the proteolytic processing of profilaggrin [6]. In addition, a deficiency of PNPLA1 induced the hyperactivation of PPARδ to increase the expression of the potential and putative target genes of PPARδ such as FA-binding protein 5 (Fabp5), small proline-rich proteins 1b (Sprr1b), and heparin-binding EGF (epidermal growth factor)-like growth factor (HB-EGF) in the epidermis [6,7]. The activation of EGF receptors controlled the proliferation and differentiation of keratinocytes [32]. The supplementation of the differentiation medium with acylceramides partially reversed the expression of these altered marker genes in *Pnpla1*-deficient keratinocytes [7]. Therefore, the loss of acylceramide due to PNPLA1 deficiency delays or disrupts the terminal differentiation and instead induces keratinocyte hyperproliferation through the EGF receptor signaling to some degree. 

## 5. Mutations of PNPLA1 Cause ARCI

Many inherited keratoses with clinical and etiologic heterogeneity follow the Mendelian model of inheritance and are classified as hereditary ichthyoses. ARCI is a subgroup of non-syndromic ichthyosis [33]. The clinical presentation and severity of ARCI may vary significantly, from the most severe and often fatal clown ichthyosis to lamellar ichthyosis (LI) and (non-herpetic) congenital ichthyosiform erythroderma (CIE) [33]. It is characterized by extensive scaling of the epidermis and a genetic defect associated with keratinization, resulting in a significantly impaired skin barrier [34]. So far, mutations in at least 14 genes have been identified to be associated with ACRI, of which the PNPLA1 gene is implicated in the pathogenesis of ARCI10 [35]. Individuals with pathogenic variants in PNPLA1 usually present at birth with pyroclastic membranes, which then transform into a CIE phenotype with scalp involvement and hyperlinear palms and soles [36].

In 2012, two homozygous mutations in the human *PNPLA1* gene were identified in ichthyosis patients for the first time [2]. Various pure and compound heterozygous mutations in the PNPLA1 gene have been identified from a registry of human ichthyosis patients. To date, approximately 59 pathogenic mutations in the PNPLA1 gene have been reported (Table 1) [2,4,24,33,34,35,36,37,38,39,40,41,42,43,44,45,46,47]. These mutations include 35 missense mutations, four code-shifting mutations, eight nonsense mutations, four deletion mutations, three splice-site mutations, two early termination mutations, and one full code mutation. Most of the mutations observed in ARCI10 patients were located within the core patatin structural domain (residues 16–185), but a small number of pathogenic mutations were located outside this structural domain. For example, a code-shifting mutation p.Ser382Alafs*74 was located outside the extended patatin structural domain and most likely affected the proline-rich structural domain (aa 335–417) [4]. We used in silico protein prediction tools including Polyphen-2 [48], SIFT [49] and PROVEAN [50] to assess the mutational deleteriousness of 59 reported PNPLA1 mutations (Table 1). Most mutations were probably damaging. In addition to humans, several mutations in the PNPLA1 gene were also demonstrated to be linked to dog ichthyosis [2,51,52]. In fact, the homozygous insertion–deletion *PNPLA1* mutations in all affected golden retrievers provided clues for the subsequent identification of human *PNPLA1* mutations in ARCI subjects [2].

How do PNPLA mutations cause ARCI in humans? The transacylase activities of the ichthyosis PNPLA1 mutants were reduced or not detected in vitro [21]. Indeed, a blockade of ω-O-acylceramide synthesis was observed in the differentiated keratinocyte in vitro and SC from PNPLA1-mutated patients [6,23]. Focal and severely attenuated CEs in SCs from PNPLA1-mutant patients displayed defective lipid coverage and were essentially composed of crosslinked proteins, indicating the impaired barrier protection of CE, whereas healthy individuals have predominantly mature hydrophobic CEs with a covalently linked outer lipid monolayer [23,42]. Massive dense hyperkeratosis, abnormal lamellar structure in the SC, and the altered release of lamellar body contents into the intercellular space at the interface between the SG and SC were observed in the skin of PNPLA1-mutant patients [42]. Moreover, PNPLA1 mutations caused LD accumulation in primary fibroblasts of ARCI patients through the impairment of both autophagosome formation and fusion of autophagosomes with lysosomes [10]. Notably, while some subjects with the same genotype exhibited consistent clinical features, there were also other subjects who showed significant variation in phenotype with the same or similar mutations [39]. 

In addition, the essential role of PNPLA1-mediated acylceramide in ACRI and the interaction of PNPLA1 with ABHD5 provided clues to elucidate the mechanisms of ichthyosis symptoms in Chanarin–Dorfman syndrome. Several mutations in ABHD5 were also demonstrated to reduce acylceramide biosynthesis catalyzed by PNPLA1 [8,9].

## 6. Concluding Remarks

Until now, PNPLA1 has been thought of as a unique transacylase essential for acylceramide biosynthesis that plays an indispensable role in the epidermal permeability barrier. As a protein specifically expressed in differentiated epidermal keratinocytes, it is of great significance to reveal the regulatory mechanism of PNPLA1 expression. Acylceramide biosynthesis is a complex process and PNPLA1 transfers the linoleoyl group from triacylglycerols to ω-OH ceramide in the final step of acylceramide biosynthesis. The main question remaining is how PNPLA1 coordinates with other enzymes to synthesize the unique acylceramide with a ULCFA moiety. Triacylglycerides are stored in LDs and ABHD5 recruits PNPLA1 to LDs. How does PNPLA1 recognize triacylglycerides with a linoleoyl group in the LDs and ω-OH ceramides with a ULCFA moiety? These questions may be answered in the future from the perspective of PNPLA1 crystal structure and the interaction and coordination of PNPLA1 with other enzymes. Several PNPLA1 mutations from ARCI patients reduced the transacylase activities of PNPAL1, inhibited acylceramide biosynthesis, and impaired CLE formation. Further revealing the molecular mechanism of ichthyosis caused by PNPLA1 mutations will thus provide new strategies to treat patients with skin barrier defects in the future. 

## Figures and Tables

**Table 1:** List of human *PNPLA1* gene mutations reported in ARCI individuals.

**Nucleotide Change ^1^**	**Aminoacid Change ^1^**	**Variation Type**	**Protein Domain**	**Reference**	**Poly-Phen-2.1 (HumVar) ^2^**	**SIFT ^3^**	PROVEAN ^4^
c.391G>T	p.Glu131*	nonsense	patatin-like	[2]	-	-	0.985
c.176C>T	p.Ala59Val	missense	patatin-like	[2]	1	0.029	0.739
c.100G>A	p.Ala34Thr	missense	patatin-like	[36]	1	0.015	0.971
c.102C>A	p.Asp34Glu	missense	patatin-like	[37]	1	0.006	-
c.387C>A	p.Asp129Glu	missense	patatin-like	[38]	1	0.012	0.924
c.56C>T	p.Ser19Leu	missense	patatin-like	[33]	1	0.001	0.989
c.514G>A	p.Asp172Asn	missense	patatin-like	[33]	1	0.007	0.999
c.421A>G	p.Lys141Glu	missense	patatin-like	[33]	0.982	0.001	0.775
c.100G>C	p.Ala34Pro	missense	patatin-like	[33]	1	0.006	0.991
c.374C>A	p.Thr125Asn	missense	patatin-like	[33]	1	0	0.999
c.488C>T	p.Pro163Leu	missense	patatin-like	[33]	1	0.038	0.999
c.266C>T	p.Pro89Leu	missense	patatin-like	[24]	0.993	0.038	0.999
c.335C>A	p.Ser112Tyr	missense	patatin-like	[24]	0.996	0.003	0.997
c.350C>T	p.Thr117Met	missense	patatin-like	[24]	1	0.001	0.999
c.418T>C	p.Ser140Pro	missense	patatin-like	[24]	0.975	0.002	0.997
c.820-820delC	p.Arg274Glyfs*7	frameshift	outside of the patatin domain	[24]	-	-	1
c.301A>G	p.Arg101Gly	missense	patatin-like	[4]	0.968	0.001	0.796
c.275delC	p.Pro92Argfs*8	frameshift	patatin-like	[4]	-	-	1
c.752C>A	p.Ala251Glu	missense	outside of the patatin domain	[4]	0.905	0	0.999
c.535C>T	p.Gln179*	nonsense	patatin-like	[4]	-	-	1
c.88G>A	p.Gly30Arg	missense	patatin-like	[4]	1	0	0.999
c.311T>C	p.Leu104Pro	missense	patatin-like	[4]	0.215	0.052	0.999
c.121delC	p.Arg41Glyfs*17	frameshift	patatin-like	[4]	-	--	1
c.667G>A	p.Glu223Lys	missense	outside of the patatin domain	[4]	0.983	0.051	0.986
c.704delC	p.Pro235Argfs*4	frameshift	outside of the patatin domain	[4]	-	-	1
c.434T>C	p.Ile145Thr	missense	patatin-like	[4]	0.986	0	0.985
c.536A>G	p.Gln179Arg	missense	patatin-like	[4]	0.999	0.279	0.982
c.158C>T	p.Ser53Leu	missense	patatin-like	[4]	1	0.009	0.989
c.496C>T	p.Arg166Cys	missense	patatin-like	[4]	1	0	0.999
c.775+3A>T	-	splice site	patatin-like	[4]	-	-	-
c.1143delC	p.Ser382Alafs*74	frameshift	outside of the patatin domain	[4]	-	-	1
c.464C>T	p.Pro155Leu	missense	patatin-like	[39]	1	0	0.999
c.92C>A	p.Ala31Asp	missense	patatin-like	[39]	0.999	0.005	0.999
c.448T>C	p.Cys150Arg	missense	patatin-like	[39]	1	0	0.999
c.1300delG;	p.Ala434fs	early termination	outside of the patatin domain	[39]	-	-	1
c.646T>C	p.Cys216Arg	missense	distal to the patatin domain	[39]	0.557	0.306	0.997
c.362A>C	p.His121Pro	missense	patatin-like	[39]	1	0.009	0.937
c.438+2C>G	-	splice site	patatin-like	[39]	-	-	-
c.939G>T c.940-952del. TGGGTTCCCAAAG	p.Glu313Dfs	early termination	outside of the patatin domain	[39]	-	-	-
c.704C>T	p.Pro235Leu	missense	distal to the patatin domain	[39]	1	0.019	0.999
c.157T>C	p.Ser53Pro	missense	patatin-like	[39]	1	0.001	0.971
c.158C>G	p.Ser53Trp	missense	patatin-like	[39]	1	0	0.999
c.733-735delTAC	p.Tyr245del	frameshift	outside of the patatin domain	[40]	-	-	0.999
c.700C>T	p.Pro234Ser	missense	outside of the patatin domain	[41]	1	0.01	0.999
c.233G>A	p.Gly78Asp	missense	patatin-like	[42]	0.966	0.206	0.579
c.527C>T	p.Thr176Met	missense	patatin-like	[42]	1	0	0.978
c.614C>T	p.Pro205Leu	missense	patatin-like	[43]	1	0.009	0.999
c.1108-1109delinsTC	p.Pro370*	Indel	outside of the patatin domain	[44]	-	-	-
c.206-1G>T	-	Splice site	patatin-like	[44]	-	-	-
c.282dup	p.Lys95*	nonsense	patatin-like	[45]	-	-	1
c.729C>G	p.Tyr243*	nonsense	outside of the patatin domain	[45]	-	-	1
c.892C>T	p.Arg298*	nonsense	outside of the patatin domain	[35]	-	-	0.999
c.417-418- delinsTC	p.Ser140Pro	missense	patatin-like	[45]	-	-	-
c.762C>G	p.Tyr254*	nonsense	outside of the patatin domain	[34]	-	-	1
c.604delC	p.Arg202Glyfs*27	frameshift	outside of the patatin domain	[46]	-	-	1
c.738-742delins	p.Gly247-Tyr248delins	in-frame	outside of the patatin domain	[46]	-	-	-
c.816dupC	p.Arg274Profs*15	frameshift	outside of the patatin domain	[46]	-	-	1
c.820dupC	P.Arg274Profs*15	frameshift	outside of the patatin domain	[46]	-	-	1
c.424delG	p.E142Rfs*26	frameshift	patatin-like	[47]	-	-	1

^1^ Reference sequences PNPLA1: NM_001145717.1, NP_001139189.2. ^2^ http://genetics.bwh.harvard.edu/pph2/ (accessed on 1 April 2022). “*” means protein translation termination. The values range from 0 to 1, with 1 being the most damaging. ^3^ http://sift.jcvi.org/www/SIFT_enst_submit.html (accessed on 17 April 2022). The values range from 0 to 1, with 0 being the most damaging. ^4^ http://provean.jcvi.org/index.php (accessed 5 May 2022) The values range from 0 to 1, with 1 being the most damaging.
